# Supplementary material for: Transcriptome Analysis Reveals Vimentin-Induced Disruption of Cell–Cell Associations Augments Breast Cancer Cell Migration
Source: Cells. 2022 Dec 13;11(24):4035. doi: 10.3390/cells11244035 (PMC9776984; doi:10.3390/cells11244035)
Supplement: Supplementary file 1 [file cells-11-04035-s001.zip › DEGs and enrichment analysis.zip!/DEGs and enrichment analysis/WT_VIMvsVector KEGG/GSEA/WT_VIM/STEROID_BIOSYNTHESIS/STEROID_BIOSYNTHESIS(HSA00100).html]

Details for gene set STEROID\_BIOSYNTHESIS(HSA00100)[GSEA]

|  || Dataset | fpkm.sample |
| Phenotype | sample.cls |
| Upregulated in class | WT\_VIM |
| GeneSet | STEROID\_BIOSYNTHESIS(HSA00100) |
| Enrichment Score (ES) | 0.7355485 |
| Normalized Enrichment Score (NES) | 1.2443626 |
| Nominal p-value | 0.0 |
| FDR q-value | 0.25000098 |
| FWER p-Value | 0.335 |
Table: GSEA Results Summary

  

Fig 1: Enrichment plot: STEROID\_BIOSYNTHESIS(HSA00100)      
 Profile of the Running ES Score & Positions of GeneSet Members on the Rank Ordered List

  

| PROBE | DESCRIPTION (from dataset) | GENE SYMBOL | GENE\_TITLE | RANK IN GENE LIST | RANK METRIC SCORE | RUNNING ES | CORE ENRICHMENT || 1 | ENSG00000242396 | AC096536.3 |  |  | 117 | Infinity | -0.0019 | Yes |
| 2 | ENSG00000167780 | SOAT2 |  |  | 2393 | 79.997 | 0.6676 | Yes |
| 3 | ENSG00000147155 | EBP |  |  | 3497 | 3.666 | 0.6813 | Yes |
| 4 | ENSG00000079459 | FDFT1 |  |  | 4414 | 2.715 | 0.6899 | Yes |
| 5 | ENSG00000172893 | DHCR7 |  |  | 5898 | 2.064 | 0.6830 | Yes |
| 6 | ENSG00000001630 | CYP51A1 |  |  | 5962 | 2.045 | 0.7001 | Yes |
| 7 | ENSG00000111012 | CYP27B1 |  |  | 6683 | 1.882 | 0.7045 | Yes |
| 8 | ENSG00000149809 | TM7SF2 |  |  | 7503 | 1.715 | 0.7059 | Yes |
| 9 | ENSG00000241095 | CYP51A1P1 |  |  | 8108 | 1.607 | 0.7099 | Yes |
| 10 | ENSG00000147383 | NSDHL |  |  | 8459 | 1.552 | 0.7177 | Yes |
| 11 | ENSG00000160285 | LSS |  |  | 10543 | 1.312 | 0.6940 | Yes |
| 12 | ENSG00000233588 | CYP51A1P2 |  |  | 10566 | 1.309 | 0.7053 | Yes |
| 13 | novel.302 | - |  |  | 10610 | 1.303 | 0.7161 | Yes |
| 14 | ENSG00000170827 | CELP |  |  | 11445 | 1.230 | 0.7128 | Yes |
| 15 | ENSG00000116133 | DHCR24 |  |  | 11668 | 1.213 | 0.7198 | Yes |
| 16 | novel.525 | - |  |  | 12491 | 1.142 | 0.7160 | Yes |
| 17 | ENSG00000132196 | HSD17B7 |  |  | 12514 | 1.140 | 0.7257 | Yes |
| 18 | ENSG00000143815 | LBR |  |  | 12529 | 1.138 | 0.7355 | Yes |
| 19 | ENSG00000104549 | SQLE |  |  | 14256 | 1.006 | 0.7152 | No |
| 20 | ENSG00000186104 | CYP2R1 |  |  | 18658 | 0.762 | 0.6475 | No |
| 21 | ENSG00000057252 | SOAT1 |  |  | 19589 | 0.710 | 0.6381 | No |
| 22 | ENSG00000052802 | MSMO1 |  |  | 20648 | 0.653 | 0.6260 | No |
| 23 | ENSG00000223901 | AP001469.1 |  |  | 21867 | 0.584 | 0.6105 | No |
| 24 | ENSG00000107798 | LIPA |  |  | 22617 | 0.536 | 0.6026 | No |
| 25 | ENSG00000099251 | HSD17B7P2 |  |  | 23382 | 0.481 | 0.5939 | No |
| 26 | ENSG00000109929 | SC5D |  |  | 23931 | 0.444 | 0.5886 | No |
| 27 | ENSG00000170835 | CEL |  |  | 24659 | 0.385 | 0.5797 | No |
| 28 | ENSG00000019186 | CYP24A1 |  |  | 25028 | 0.349 | 0.5765 | No |
| 29 | ENSG00000240720 | LRRD1 |  |  | 30151 | 0.000 | 0.4899 | No |
| 30 | ENSG00000215223 | CYP51A1P3 |  |  | 42291 | NaN | 0.2846 | No |
| 31 | ENSG00000230739 | AL392003.2 |  |  | 45508 | NaN | 0.2303 | No |
| 32 | ENSG00000232908 | HSD17B7P1 |  |  | 47696 | NaN | 0.1933 | No |
| 33 | ENSG00000224339 | AL121578.1 |  |  | 49202 | NaN | 0.1680 | No |
| 34 | ENSG00000250229 | AC111193.1 |  |  | 57459 | NaN | 0.0284 | No |
Table: GSEA details [plain text format]

  

Fig 2: STEROID\_BIOSYNTHESIS(HSA00100)      
 Blue-Pink O' Gram in the Space of the Analyzed GeneSet

  

Fig 3: STEROID\_BIOSYNTHESIS(HSA00100): Random ES distribution      
 Gene set null distribution of ES for **STEROID\_BIOSYNTHESIS(HSA00100)**
